# Mechanisms and Materials of Flexible and Stretchable Skin Sensors

**DOI:** 10.3390/mi8030069

**Published:** 2017-02-28

**Authors:** Yicong Zhao, Xian Huang

**Affiliations:** Biomedical Engineering, School of Precision Instrument and Opto-Electronics Engineering, Tianjin University, Tianjin 300072, China; zhaoyicong@tju.edu.cn

**Keywords:** flexible electronics, stretchable electronics, skin sensors, precision medicine, health monitoring, wearable technology

## Abstract

Wearable technology has attracted significant public attention and has generated huge societal and economic impact, leading to changes of both personal lifestyles and formats of healthcare. An important type of devices in wearable technology is flexible and stretchable skin sensors used primarily for biophysiological signal sensing and biomolecule analysis on skin. These sensors offer mechanical compatibility to human skin and maximum compliance to skin morphology and motion, demonstrating great potential as promising alternatives to current wearable electronic devices based on rigid substrates and packages. The mechanisms behind the design and applications of these sensors are numerous, involving profound knowledge about the physical and chemical properties of the sensors and the skin. The corresponding materials are diverse, featuring thin elastic films and unique stretchable structures based on traditional hard or ductile materials. In addition, the fabrication techniques that range from complementary metal-oxide semiconductor (CMOS) fabrication to innovative additive manufacturing have led to various sensor formats. This paper reviews mechanisms, materials, fabrication techniques, and representative applications of flexible and stretchable skin sensors, and provides perspective of future trends of the sensors in improving biomedical sensing, human machine interfacing, and quality of life.

## 1. Introduction

Rapid growth in electronic technology yields miniaturized electronic devices and recent evolution of wearable electronic technology that can be integrated on human bodies and conduct diverse functions, such as mobile computation [1], health monitoring [2,3], activity tracking [4,5,6], and rehabilitation [7]. Wearable electronic devices can combine with portable electronic gadgets such as cell phones, laptops, and tablets to offer access to remote resources and enable data exchange, analysis and diagnosis. The wearable devices demonstrated both by various commercial available devices [8] as well as devices under exploration [9] have shown great promise to enrich personal health records and facilitate biomedical informatics, both of which are considered essential elements in the newly proposed precision medicine [10,11]. However, current wearable devices are predominately realized by encapsulating integrated circuits on solid substrates in rigid packages, which are mechanically incompatible with soft and curvilinear human body, resulting in unreliable and unrepeatable measurement results due to unreliable skin contact and changing measurement locations.

Some wearable devices are based on flexible and stretchable skin sensors, which are used primarily for biophysiological signal sensing and biomolecule analyzing on skin. These sensors can serve as activity tracking devices to record basic biophysiological parameters, or used for facilitating diagnosis and treatment of certain diseases such as diabetes [12], cystic fibrosis [13], dermatitis [14,15], and peripheral vascular disease [16]. In addition, they can be used as human–computer interface to assist human with speech and action disorders [17,18]. Additional use of skin sensors may involve monitoring exogenous parameters such as air qualities, environmental temperature, ultraviolet (UV) exposure, and humidity, allowing comprehensive assessment of health-related issues by considering both environmental and internal effects. The skin sensors offer mechanical compatibility to human skin and maximum compliance to skin morphology and motion. Stretchability is essential for these skin sensors, as the sensing precision, repeatability, stability and adhesion to skin are all determined by the capability of the sensors in following the skin motion, which causes skin deformation up to 30% [19]. The skin sensors contain unique structures constructed by either intrinsically soft materials or thin film materials on elastomer substrates. They can be simply mounted on bodies using fixtures such as bandages and body straps or use improved approaches that allow spontaneous skin attachment by van der Waals force using ultrathin and soft materials [14,18,20]. In addition, pressure sensitive silicon adhesive [21,22] can also be used to enhance the interface between the sensors and the skin, and offer reversible adhesion for long-term skin integration. Although the underlying mechanisms and relevant techniques of the skin sensors have been studied in many research papers that focus on various aspects [20,23,24], it will be beneficial if a systematic summary can be offered with comprehensive review of the state-of-the-art technology in flexible and stretchable skin sensor development.

This paper reviews some essential elements of flexible and stretchable skin sensors, including their mechanisms, materials, fabrication techniques, and applications, all of which represent recent progress in both theoretical and applied research of skin sensors. The fundamental mechanisms that determine the stretchability of the sensors are first presented, followed by materials used in skin sensors and their processing techniques. Finally, representative applications of the skin sensors are presented to demonstrate their capability in the areas of biomedical sensing and daily activity tracking. Flexible and stretchable skin sensors hold the promise to replace current wearable sensors based on rigid substrates and packages, and may eventually lead to the revolutionary changes in the formats of continuous, long-term health monitoring devices to improve social health levels.

## 2. Mechanisms of Flexible and Stretchable Skin Sensors

Flexible and stretchable skin sensors offer maximum compliance to the skin, and, thus, minimum reaction force from the sensor in response to the deformation, allowing less influence to the normal functions of the skin. The stretchability of the skin sensors can be achieved at the structural and material levels. The former refers to unique designed structures that offer tolerance to certain levels of deformation within the limits of the fracture strain of the constituent materials, while the latter can be attributed to intrinsically soft materials that are mechanically elastic to allow reversible extension and compression in response to external forces. The following section summarizes details of these two approaches.

### 2.1. Stretchable Structures

Materials used in skin sensors follow a simply rule that the bending strain of the materials decreases linearly with thickness of materials [23]. As a result, composition materials of skin sensors such as semiconductors, polymers, and metals are used in formats of ribbons, wires, and membranes with thickness in the scale of tens of nanometers to a few micrometers. They can be readily bended to reach a radius of curvature of ~150 mm with ~0.1% strains [25,26], which is less than the facture strain of these materials [26]. Two design approaches have been developed to make intrinsically rigid materials stretchable on elastomeric substrates. The former design uses out-of-plane buckling of ultra-thin nanoscale wires, ribbons, or membranes to release stress caused by in-plane prestrain applied to the substrates. The latter uses stretchable interconnects as bridges to connect with rigid islands, which typically contain functional components such as sensors [27,28], electronics [29,30,31], and commercial off-the-shelf components [32]. Both of these strategies have been widely used in layout of stretchable skin sensors.

#### 2.1.1. Out-of-Plane Design

**Figure 1:**
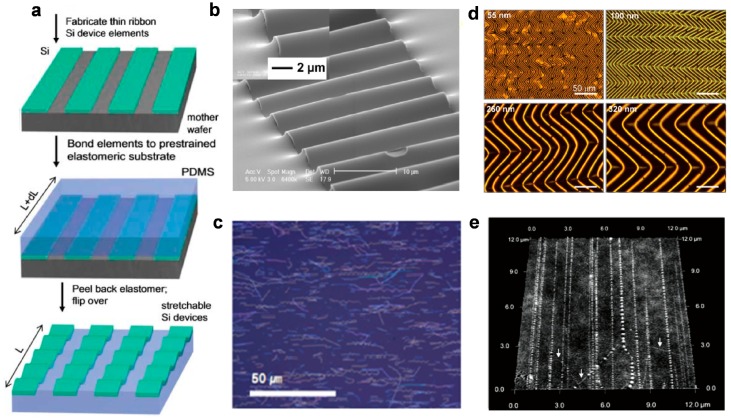
Mechanism of out-of-plane stretchable structures: (**a**) formation of out-of-plane nanoribbons (Reprinted with permission from Ref. [30] Copyright 2006 American Association for the Advancement of Science); (**b**) scanning electron microscope (SEM) images of a wavy nanoribbon (Reprinted with permission from Ref. [24] Copyright 2010 American Association for the Advancement of Science); (**c**) a large area optical micrograph of silicon nanowires (Reprinted with permission from Ref. [39] Copyright 2009 American Chemical Society); (**d**) optical micrographs of 2D wavy Si nanomembranes with various thickness (55, 100, 260, 320 nm) on polydimethylsiloxane (PDMS), formed with a thermal prestrain of 3.8% (Reprinted with permission from Ref. [40] Copyright 2007, American Chemical Society); and (**e**) an atomic force microscopic image of wavy SWNTs on a PDMS substrate (Reprinted with permission from Ref. [34] Copyright 2008 American Chemical Society).

The formation of out-of-plane design with buckling structure is illustrated in Figure 1a. The ultrathin ribbons can be fabricated using conventional lithography process, followed by bonding the nanoribbons on to a prestrained elastomeric substrate. Releasing of the prestrain leads to periodical wavy structures on both ribbons and the substrate. The wavelength (λ) and magnitude (*A*) of the wavy structures can be determined by following equation.

(1) $$\lambda =2\pi h_{f}{\left(\frac{{\bar{E}}_{f}}{3{\bar{E}}_{s}}\right)}^{1/3},A=h_{f}\sqrt{\frac{\varepsilon_{\mathrm{pre}}-\varepsilon_{\mathrm{applied}}}{\varepsilon_{c}}-1}$$

in which $h_{f}$ is the thickness of the stiff ribbons, $\bar{E_{f}}$ the Young’s modulus of the elastic substrate, $\varepsilon_{\mathrm{applied}}$ the applied strain, $\varepsilon_{\mathrm{pre}}$ the prestrain level, and $\varepsilon_{c}$ the critical strain for the buckled ribbons. The peak strain in the ribbon is approximately equal to

(2) $$\varepsilon_{\mathrm{peak}}\approx 2\sqrt{\left(\varepsilon_{\mathrm{pre}}-\varepsilon_{\mathrm{applied}}\right)\varepsilon_{c}}$$

As a result, the maximum stretchability of the wavy structures can be determined by equating $\varepsilon_{\mathrm{peak}}$ to facture strain of the ribbon materials. 

**Figure 2:**
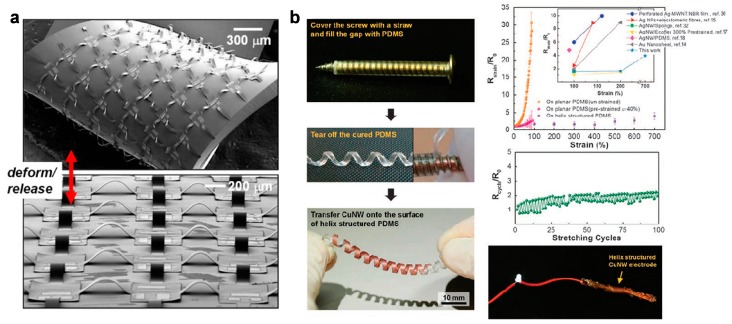
Examples of out-of-plane strcutres with high stretchability: (**a**) SEM images of arrays of complementary metal-oxide semiconductor (CMOS) inverters with spatially buckled bridges (Reprinted with permission from Ref. [29] Copyright 2008 National Academy of Sciences); and (**b**) helical-structured copper nanowire (CuNW)-based electrodes (Reprinted with permission from Ref. [41] Copyright 2014 Nature Publishing Group).

**Figure 3:**
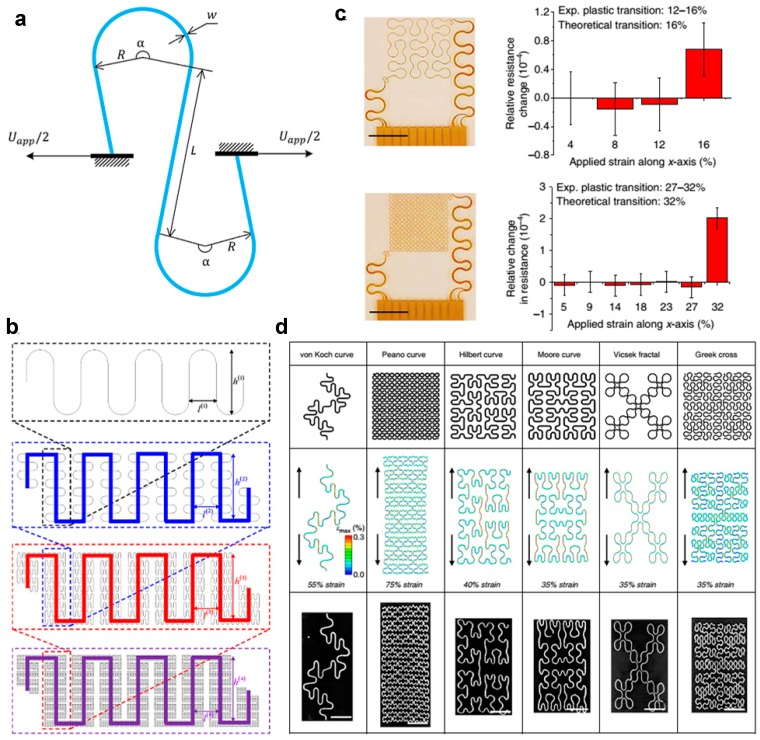
Mechanisms of serpentine and fractal structures: (**a**) a serpentine interconnect subjected to an axial stretching (*U*_app_) at the two ends (Reprinted with permission from Ref. [47] Copyright 2016 Elsevier); (**b**) schematic illustration on the geometric construction of self-similar serpentine interconnects (Reprinted with permission from Ref. [48] Copyright 2013 Elsevier); and (**c**,**d**) representatives of fractal structures (Reprinted with permission from Ref. [45] Copyright 2014 Nature Publishing Group).

Recent development of wavy design has led to more complicated three-dimensional (3D) structures that buckle at higher orders, indicating potential applications of these technologies to form miniaturized flexible and stretchable electronics with highly spatial complexity and capability to achieve predefined shape shifting and function alternation [33]. The wavy design has been wildly available to integrate silicon [24], carbon nanotubes [34,35,36], graphene [37] and ferroelectrics [38] in formats of nanoribbons (Figure 1b) [24], nanowires (Figure 1c,e) [39], and nanomembranes (Figure 1d) [40]. However, the stretchability of the wavy design is determined by the prestrain levels of the substrates and the bending curvature of the materials as shown in Equations (1) and (2), limiting the applications of this technology in situations that require larger stretchability and less complex fabrication processes. As a result, island–bridge configurations have been developed to offer improved stretchability with both out-of-plane and in-plane structures in which islands based on functional sensing and circuit elements are mechanically and electrically connected with bridges made of narrow polymeric and metallic strips. The bridges, which contain either straight or serpentine interconnects, are freely suspended or bond on substrates between two islands. The stretchability of such structures are achieved either by deformation of the spatially buckled bridges (Figure 2a) or planar deformation of the interconnects (Figure 3c).

Novel substrate-free spatial helical structures have also been developed using diverse twisting modes, and have been used to act as sensors and power harvesters. Shang et al. have explored a carbon nanotube (CNT) yarn supercapacitor utilizing the helical loop structure. The entire structure can withstand strain of ~150% and repeated high frequency stretching (up to 10 Hz over 10,000 cycles). A helical spring based on copper nanowire [41], as shown in Figure 2b, offers higher stretchability (~700%). Similar structures can be formed by other metallic nanowires, showing variety of potential applications in wearable sensors and interconnects that can deform with the gradual growth of body parts.

#### 2.1.2. In-Plane Design

Improved deign without using prestrained substrates can be achieved by in-plane island–bridge design. The polymeric and metallic interconnects are typically in forms of serpentine or fractal [42,43,44,45,46] meshes that are completely bonded onto elastomeric substrates. In some cases, the island structures can be further omitted, resulting in continuous self-similar serpentine or fractal structures as both sensors and interconnects. Compared to wavy structures, these planar serpentine or fractal design effectively accommodate much larger applied strain through in-plane structural deformation without requirement of prestrain on substrates, and eliminate the concern of delicate spatially-buckled structures that can be easily broken under external scratch.

However, design of serpentine interconnect is still largely empirical, only a few theoretical models have been developed to analyze the deformation and stretchability of serpentine geometry. Fan et al. formulated an analytic model of in-planar serpentine interconnects based on finite deformation theory [47]. As illustrated in Figure 3a, a serpentine interconnect is simplified as three straight wires with length *L* or *L/2* connected with two arcs with an identical radius *R* and an arc angle α. Three dimensionless parameters, width/radius ratio $\bar{w}=$ *w*/*R*, arm length/radius ratio $\bar{L}=$ *L*/*R* and arc angle α, can then be used to represent the shape of the serpentine interconnect. As $\bar{w}$ of the non-buckled interconnect is usually much smaller than 0.5, such interconnect can be modeled as a curved, Euler–Bernoulli beam. When the serpentine interconnect is subjected to a tensile displacement of $U_{\mathrm{app}}/2$ at the end, the effective applied strain $\varepsilon_{\mathrm{app}}$ of the serpentine interconnect can be represented by

(3) $$\varepsilon_{\mathrm{app}}=\frac{U_{\mathrm{app}}}{4R\sin\left(\alpha /2\right)+2L\cos\left(\alpha /2\right)}$$

The peak value of maximum principal strain in the serpentine interconnect can be related to the applied strain and the geometric parameters by

(4) $$\varepsilon_{\max-\mathrm{nonlinear}}=\bar{w}F_{2}\left(\bar{L},\alpha ,\varepsilon_{\mathrm{app}}\right)$$

where $F_{2}\left(\bar{L},\alpha ,\varepsilon_{\mathrm{app}}\right)$ is a function that can be determined numerically using an approximate model based on finite deformation theory.

A fractal design concept that allows formation of stretchable layouts through stepwise iterations of basic shape units has been introduced to realize highly stretchable lithium-ion batteries [43] as well as several epidermal sensors [31,44,49]. As illustrated in Figure 3b, a fractal-based layout is created from the first order of serpentine geometry, and then constructed by connecting multiple copies of the unit cell forming self-similar design that offers increased area coverage and improved stretchability [48]. Theoretical models have been developed to analyze the deformation and stretchability of the fractal geometry. Fan et al. [45] have studied the deformations of various fractal layouts (Figure 3d), using a finite element method (FEM) followed by experimental evaluation. They also introduced a high precision approach to measure the elastic-plastic transition (or the elastic stretchability) through measuring differential resistances of the fractal interconnects, showing reasonable consistency with numerical analysis (Figure 3c). Several analytical models have been developed to determine elasticity for fractal interconnects. For example, Zhang et al. [48] have developed analytical models of flexibility and elastic stretchability, through establishing recursive formulae at different fractal orders. The analytical models show that the stretchability of system increases with the order of self-similar interconnect, and a surface filling ratios of 50% would yield 70% stretchability. In addition, the tensile stiffness for fractal interconnects has been determined by analytic approach, and has been verified by finite element analysis and experiments [50].

### 2.2. Intrinsic Elastic Materials

The stretchability of skin sensors can be achieved at the material levels using intrinsic elastic materials. Major materials in this category include elastomers and liquid metals, all of which adopt the stretchability due either to the flexible and long polymer chains or to weak intermolecular forces. This section offers a general review of the mechanism of these materials, while a detailed list of these materials will be given in next section.

One of the most widely used materials in stretchable skin sensors is silicone-based elastomers represented by polydimethylsiloxane (PDMS), fluorosilicone and various commercially available products under different tradenames such as Ecoflex, Dragon Skin, and Solaris. Silicone-based elastomers are notable for their high electrical resistivity (e.g., 2.9 × 10^14^ Ω·cm for PDMS), low glass transition temperatures (e.g., −125 °C for PDMS), large thermal coefficient of expansion (typically 4.8 × 10^−4^ K^−1^) and high flexibility (with Young’s module of 1 MPa). The polymer chains of the silicone contain siloxane backbones that consist of alterative sequences of silicon and oxygen and two organic substituents connected to each silicon atom, resulting in various properties (e.g., chemical resistance, elasticity, and phase) and processibility (e.g., curing time, and curing temperature). The elastomers obtain their stretchability through highly flexible siloxane backbones, which can be stretched under external forces. Another material that widely used in stretchable skin sensors is polyurethane (PU), which contains urethane groups connected with other groups such as ester, ether, amine and urea. The PU elastomers adopt their elasticity from the elastic polyol parts in the polymer chains, and offer large tear strength and abrasion resistant than silicone rubbers, making them ideal materials to construct substrates for skin sensors when frequent surface scratch and impact are expected.

## 3. Materials in Skin Sensors

The major materials of stretchable skin sensors can be classified into two categories. One involves various intrinsically stretchable materials, such as elastomers, liquid metals, and composite materials. The other includes materials such as solid metals, semiconductors, polymers, and inorganic compounds, which are rigid as bulk materials, but can be used as ultrathin films or membranes designed into special stretchable structures with thickness ranging from tens of nanometers to tens of micrometers. Therefore, the Young’s modulus of the materials for stretchable skin sensors are wildly distributed from 0 to 10^12^ Pa [51].

### 3.1. Physically Soft and Stretchable Materials

#### 3.1.1. Elastomers

Elastomers are available in different compositions with varied stretchability. As the fundamental materials in stretchable skin sensors, elastomers are mainly used as substrates, binders and adhesion layers. Among the available elastomers, PDMS is most commonly used. The mechanical properties of PDMS can be tuned by varying the curing conditions such as chemical ratios, temperature, and time, resulting in a Young’s modulus in a controllable range from 1 to 150 MPa, and a stretchability up to 100%. By alternating the side groups as well as the lengths of the polymer chains, it is possible to obtain different types of elastomers with different physical and chemical properties. For example, PU and acrylic elastomer are two alternatives for skin sensor substrates, and are softer than PDMS due to its low Young’s modules. The maximum stretchability that can be achieved is 300% [52]. The low-temperature curing silicone is a type of adhesive that can provide pressure sensitive reversible bonding between the skin and the devices. Besides of mold casting, silicone such as PDMS and polyurethane acrylate (PUA) can be photocurable to allow pattern definition through traditional photolithography processes [53,54] or even 3D printing techniques [55].

#### 3.1.2. Liquid Metals

Liquid metals such as eutectic gallium-indium (eGaIn) and gallium-indium-tin (Galinstan) are intrinsically elastic with low resistivity (~2.9 × 10^−7^ Ω·m), low viscosity (~2 × 10^−3^ Pa·s), and low toxicity [56]. Their melting points are 15.5 °C [57] (75 wt % Ga and 25 wt % In) and −19 °C [58] (68.5 wt % Ga, 21.5% In, and 10.0% Sn), respectively, resulting in their liquid states at room temperature. Various functional components such as pressure sensors [59,60], strain sensors [61], antennas [62,63], and soft wires [64] have been fabricated by injecting liquid metals into microfluidic channels. Devices made of liquid metals can withstand deformation of microchannels at very high strain (up to 800%) [65].

#### 3.1.3. Conductive Polymers

Conductive polymers (CPs) applied in stretchable electronics can be achieved by intrinsically conductive polymers (ICPs) [66] or conductive polymer composites [67]. Intrinsically conductive polymer materials such as synthetic poly(acetylene) (PA), poly(pyrrole) (PPy), poly(thiophene) (PT), poly(aniline) (PANI), and poly-(3,4-ethylenedioxythiophene) (PEDOT) can be realized by conjugation of polymer backbone, forming high energy orbitals with loosely bonded electrons to corresponding atoms, allowing maximum facture strain at the level of 1000% [68]. The conductive polymer composites are composed of polymers and conductive fillers (e.g., metal nanoparticles, metal nanowires, graphite, carbon nanotubes, and graphene). Conductive polymers are subject to influence of strain, which may lead to increased resistivity with strain. Park et al. have demonstrated a conductive composite mat using electrospun poly(styrene-block-butadiene-blocks-tyrene) (SBS) rubber fibers embedded with silver nanoparticles, leading to high conductivity even under large deformations (σ ≈ 2200 S·cm^−1^ at 100% strain) [69]. Shang et al. have achieved an elastic conductive nanocomposite composed of multiwall carbon nanotubes (MWNTs) and polyurethane (PU), which has initial conductivity of more than 5.3 S·cm^−1^ and stretchability of more than 100% [70]. Niu et al. have realized buckled single-wall carbon nanotube (SWCNT) electrodes by fabricating directly grown SWCNT films with continuous reticulate architecture on pre-strained PDMS [71]. The electrodes can stretch under a strain of 140% without significant change of resistance.

#### 3.1.4. 1D and 2D Materials

The applications of one-dimensional (1D) and two-dimensional (2D) materials to construct stretchable electronics represent important trends in constructing stretchable electronics. Representative 1D and 2D materials include multi-walled or single-walled carbon nanotubes [72,73], silicon nanowires [74], metal nanowires [75,76], graphene [77], and transition metal dichalcogenides (TMDCs) [78]. Among them, both carbon nanotubes and graphene possess high electron mobility (~10^5^ cm^2^·v^−1^·s^−1^ for carbon nanotube [79] and ~2 × 10^5^ cm^2^·v^−1^·s^−1^ for graphene [80] at room temperature) and excellent mechanical flexibility (~1 Tpa Young’s modulus) [81], making them promising materials for high performance electronic devices, such as top-gated transistors [82,83,84]. When used as sensors, the large surface-to-volume ratios of the 1D and 2D materials can lead to improved capabilities, such as highly sensitive biochemical sensing and large interfacial adhesion [85]. In addition, the optical transparency of graphene and carbon nanotubes allows construction of fully transparent sensors that possess high flexibility and softness [86,87]. Some excellent reviews about the 1D and 2D materials used in flexible and stretchable electronics have been provided by the following articles [88,89,90].

### 3.2. Unique Stretchable Structures

As mentioned in the previous section, raw materials that are rigid in their bulky formats can also offer stretchability in ultrathin configurations and unique stretchable design. Some major materials used for constructing skin sensors include metals, semiconductors, polymers, and inorganic compounds.

#### 3.2.1. Solid Metals

Solid metals are intrinsically hard conductive materials that would become flexible when appear as thin films. Dominant metals used in skin sensors include Au, Cu, Al, Cr, Ti and Pt, which are used for conductive interconnects, electrodes, sensors, contact pads and other circuit components (e.g., resistor, inductors, and capacitors). These metals are typically tens of nanometers to a few micrometers in thickness, and are deposited on target substrates through physical deposition, electrochemical plating, and direct printing approaches. Many of these metals are ductile with a fracture strain of less than 1%. However, the stretchability of the metallic thin films can reach more than 100% when designed into special formats such as self-similar serpentine [91], fractal [28], helical [92] and prestrained bulking [29].

#### 3.2.2. Semiconductors

Various active components such as diodes, transistors, and light emitting diodes (LED) can be made of inorganic semiconductor materials (e.g., silicon [30], GaAs [93], ZnO [94], InP [95], GaN [96]) as well as organic semiconductor materials (e.g., poly(3-hexylthiophene) (P3HT) [97], Poly(p-phenylene)vinylene [98], and Poly(2,5-bis(3-hexadecylthiophen-2-yl)thieno[3,2-b]thiophene) (pBTTT)) [99]. The bending stiffness and bending-induced strain of these rigid semiconductor materials can be exceptionally small due to cubic and linear scaling of these quantities with thickness of the materials. These semiconductor materials can be patterned into nanomembranes [100], nanoribbons [101], and nanowires [95] through complementary metal-oxide semiconductor (CMOS) fabrication processes.

#### 3.2.3. Polymers

Polymers offer mechanical and electrical supports to skin sensors. They can be used as structural layers, electrical insulation layers, and dielectric layers in the skin sensors. Many polymers have been used to construct skin sensors, including some most prominent ones such as polyimide, poly(methyl methacrylate) (PMMA) and parylene. These polymers offer high mechanical strength that are ideal as structural layers to support the skin sensors. In addition, these polymers are typically thermal setting materials that can be easily obtained through spin-coating and dipping followed by curing at escalated temperature. Parylene is an excellent dielectric material, its fabrication process involves chemical vapor deposition (CVD), allowing pinhole-free uniform layers on curved or irregular surface. 

## 4. Fabrication Techniques

Fabrication of skin sensors involve a series of techniques that combine conventional fabrication methods such as microelectromechanical systems (MEMS) technology, CMOS process, and mechanical milling with emerging techniques such as printable electronic, additive manufacturing, and laser process. Under the support from diverse fabrication techniques, many materials can be processed to yield structures in stretchable skin sensors.

### 4.1. Conventional Microfabrication Processes

**Figure 4:**
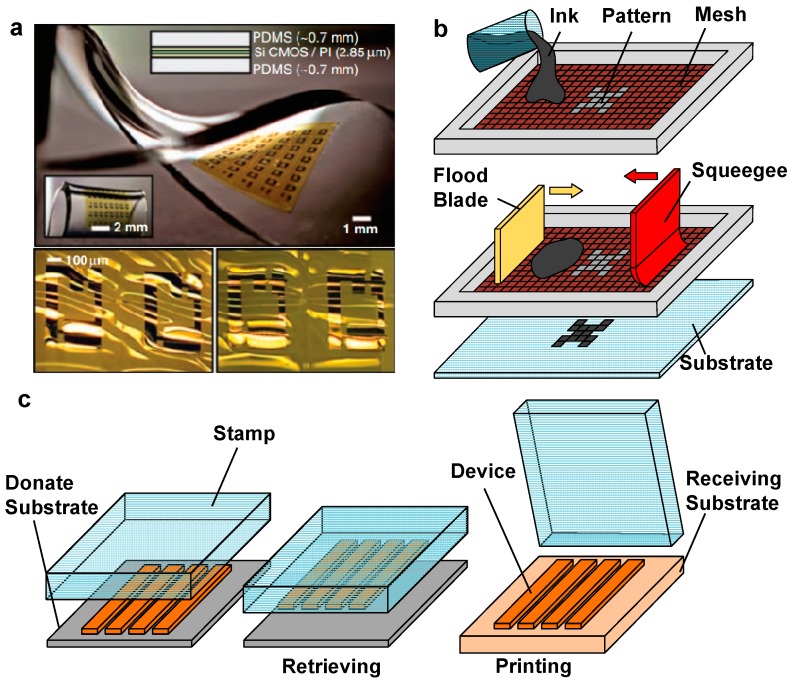
(**a**) Stretchable integrated circuits fabricated based on CMOS technology (Reprinted with permission from Ref. [26] Copyright 2008 American Association for the Advancement of Science); (**b**) a schematics of the screen printing process; and (**c**) a schematics of the transfer printing process.

Fabrications of active and passive components in flexible and stretchable electronic skin sensors can be achieved by MEMS and CMOS technology. The fundamental challenge of using MEMS and CMOS technology to make skin sensors involve application of ultrathin membranes as compared with rigid or brittle materials used in traditional MEMS and CMOS fabrication processes. Processes for fabricating flexible and stretchable skin sensors can be categorized into device-last and device-first approaches. The former involves fabricating active components on silicon membranes on silicon-on-insulator substrates, and then transfer-printing the membranes to destination substrates for further processing. While the latter refers to thinning down a thick semiconductor substrates integrated with functional components using physical sanding or chemical etching methods. The device-last approaches have been described by several research works. For example, Kim et al. [26] have developed stretchable integrated circuits, which combine multilayer neutral mechanical plane layouts and “wavy” structural configurations, including logic gates, ring oscillators, and differential amplifiers on silicon nanomembrane with thickness of 250 nm (Figure 4a). The device-first approaches have been demonstrated by MOS capacitors [102,103], memory cells [104,105], batteries [106], and MEMS switches [107].

Simplified approaches for making active and passive components involve using both organic materials as conductors, semiconductors, and dielectric to construct electronic devices. The major fabrication processes include chemical or physical vapor deposition, spin-coating and dip coating, which are much convenient than conventional microfabrication processes of inorganic materials, whose fabrications typically require thermal diffusion, ion implantation, and highly corrosive acid etchant used in inorganic materials. However, drawbacks of using organic materials are their low conductivity (1000 S·cm^−1^) and low charge mobility (approximately 10^−2^ to 10^2^ cm^2^·v^−1^·s^−1^) [108,109], which hinder the applications of organic materials in high performance electronics.

### 4.2. Printable Electronics

Continuous development of printing electronic technology enables the application of such technology in developing flexible and stretchable electronics. Printable electronic technology allows direct generation of patterns on soft substrates through additive manufacturing technology such as screen-printing, slot-die coating and inkjet printing. In addition, a special printing approach used primarily in flexible and stretchable electronics is based on transfer printing, which transfer components fabricated on donate substrates onto target substrates that are typically thin plastic films, metal foils, and elastomer membranes. 

#### 4.2.1. Screen Printing

Screen printing is a low-cost, high-throughput printing technique used to construct skin sensors. The working principle of screen printing system is illustrated in Figure 4b. A screen printing system typically contains a flood blade that moves across a screen with open meshes with pore sizes ranging from 10 to 200 μm [110,111] and fills the meshes with ink. A squeegee is then moved in an opposite direction to push the inks in the meshes towards the substrates. Eventually, the adhesion force from the substrates pulls the inks down in a close distance, resulting in pattern formation that is determined both by the properties of the inks and the size of the meshes. Screen printing is notable for its low fabrication cost and capability to print single or stacked layers onto variety of soft materials such as fabrics and plastic films. The ability of screen printing technique has been demonstrated majorly in the area of printing organic devices such as organic light-emitting diode (OLEDs) [112], organic field-effect transistor (OFETs) [113,114], thin film batteries [115,116,117], and organic solar cells [118,119]. In addition, it has been widely used to fabrication various flexible and stretchable electronic components, including antennas [120], metal conductors [121,122], and thin-film transistors [123,124,125]. Limitations of screen printing include limited selection of ink materials, short processing time influenced by solvent evaporation, and low printing resolution (>10 µm).

#### 4.2.2. Inkjet Printing

Inkjet printing is considered as an additive manufacturing technique that is attractive for making electronic components on flexible substrates without using any photomask. Inks that contain either fully dissolved chemicals [126,127] or nanoparticles [128,129,130,131] can be deposited through inkjet nozzles, which are actuated through a number of mechanisms such as piezoelectricity and aerosol. Post sintering processing after inkjet printing using direct heating, microwave, laser, and pulsed light can enhance the performance of the printed patterns by converting individual nanoparticles into connected matrixes. Inkjet printing techniques have been used to fabricate various electronic devices. Passive components such as resistors [132,133,134], capacitors [135,136,137] and inductors [138] have been printed on polymer substrates with various functional inks. Active components such as thin film transistors [139,140,141] and LED [142,143,144] have also been fabricated using inkjet printing methods. Development of colloidal solution for proper ejection of droplets on a targeted area by keeping an acceptable quality of the printed circuits is challenging due to the influence of evaporation rate of the solvents and orientation of the active particles. Despite the advancement of both control electronics and nozzle technology in inkjet printing, its printing speed (at the scale of 10 mm·s^−1^) are still low as compared with screen printing methods, and its capability in printing complex structures such as serpentine and meander is still demanding further improvement. In addition, possibility of nozzle clogging and limited numbers of nozzles that can work simultaneously making inkjet printing methods more a rapid prototyping tool in labs rather than an acceptable mass fabrication method for industry. The spreading of the printed ink on target substrates and chaotic behavior of droplets during the time of flight further add to the issues of inkjet systems.

#### 4.2.3. Transfer Printing

Transfer printing is an essential procedure to obtain flexible CMOS/MEMS devices. The target devices can be firstly fabricated on a donate substrate and then transferred to a receiving substrate using a viscoelastic stamp (usually a PDMS stamp) (Figure 4c). In this process, the adhesive strength is directly proportional to separation speed of the stamp from a surface. The effective separation speed between the stamp and the substrates is approximately 10 cm·s^−1^ or greater during a retrieval process, and is a few mm·s^−1^ or less for the printing process. Transfer printing technique has been extensively exploited to assemble diverse classes of materials (e.g., semiconductors, metals, carbon, and organic), thereby providing an effective method to fabricate various devices ranging from simple light emitting diodes [145], transistors [146,147] and sensor elements to fully integrated circuits [148].

## 5. Applications of Flexible and Stretchable Skin Sensors

Mechanisms, materials and fabrication approaches mentioned above can be used to construct diverse skin sensors that offer broad applications in health monitoring, daily activity tracking, and rehabilitation. These skin sensors have been used to record biophysical signals such as biopotential, skin strain, temperature, and hydration. In addition, initial efforts have been made to conduct specific biomolecule analysis using body fluids (e.g., sweat and blood) through direct contact or transdermal sensing approaches.

### 5.1. Biopotential Measurement

**Figure 5:**
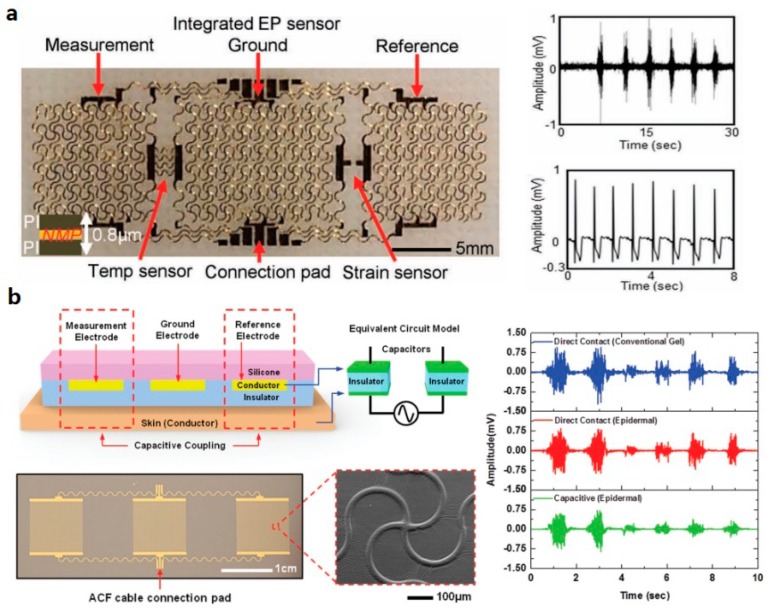
Examples of skin sensors for biopotential measurement: (**a**) biopotential measurement using skin-contacted metallic electrodes (Reprinted with permission from Ref. [91] Copyright 2013 John Wiley and Sons); and (**b**) an epidermal electronic system (EES) with a capacitive sensor for electrophysiological (EP) measurement (Reprinted with permission from Ref. [18] Copyright 2014 John Wiley and Sons).

Biopotential measurement using skin sensors represents one of the most important applications of skin sensors. Due to the capability to be mounted on different locations of human skin, electrodes based on stretchable conductive meshes typically made of copper and gold have been used to conduct electroencephalogram (EEG), electrooculogram (EOG), and electrocardiogram (ECG) measurements on body. Yeo et al. [149] have introduced a multifunctional epidermal electronic systems measuring electrophysiological based on skin-contacted metallic electrodes or meshes made of gold and copper, which can measure ECG, EMG, temperature and strain (Figure 5a). Due to close skin contact, the flexible and stretchable skin electrodes can directly contact with skin with a contact resistance at a scale of 35 kΩ, which is compared smaller than conventional dry electrodes (~40 kΩ). Non-contact biopotential sensing is also feasible. Jeong et al. have demonstrated capacitive electrodes that can measure biopotential signals without direct contact with skin [18] (Figure 5b). They have also presented another skin sensor that measures EMG signal induced by arm and wrist movement to control unman drone.

### 5.2. Strain Sensing

**Figure 6:**
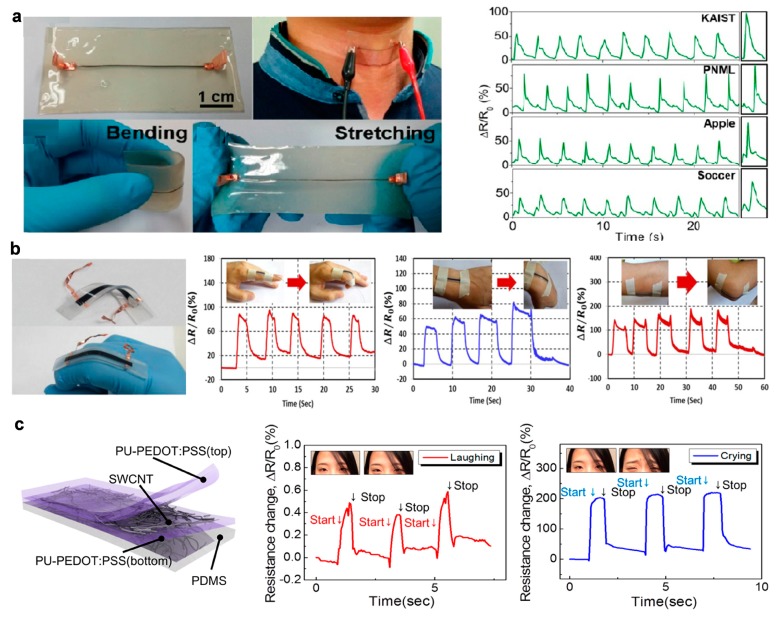
Examples of skin sensors for strain sensing: (**a**) graphene strain sensor embedded in an elastomeric patch that is bendable and stretchable, for detection of the motions of throat (Reprinted with permission from Ref. [17] Copyright 2015 American Chemical Society); (**b**) application of the CNT–Ecoflex nanocomposite based strain sensors to human motion detection (Reprinted with permission from Ref. [150] Copyright 2015 IOP Publishing); and (**c**) a transparent strain sensor consisting of three-layer stacked nanohybrid structure of PU-PEDOT:PSS/SWCNT/PU-PEDOT:PSS on a PDMS substrate (Reprinted with permission from Ref. [87] Copyright 2015 American Chemical Society).

Strain sensors can be directly attached on skin to measure strain induced by skin deformations caused by respiration, heartbeat, bending of body joints and muscle activities. Strain sensors can be fabricated by diverse materials and methods [17,150,151] with applications ranging from personalized health-monitoring [152,153] to human-machine interfaces [154,155] and soft robotics [156,157,158]. Park et al. [17] have achieved a stretchable graphene strain sensor using a layer-by-layer assembly method, in which stretchable yarns are repeatedly dip-coated with poly(vinyl alcohol) and graphene nanoplatelets bilayer. This strain sensor can be attached to throat and monitors the motions caused by speaking with maximum stretchability of ~100% (Figure 6a). Surface matrixes made of carbon nanotube and silicone rubber have been used to make strain sensors (Figure 6b). Carbon nanotubes were first coated onto a patterned polyimide film through air spraying, and were then transferred onto an Ecoflex film by casting uncured Ecoflex onto a polyimide film. The carbon nanotubes and Ecoflex form surface matrix that can be separated from the polyimide after the curing of the Ecoflex, resulting in conductive composite with high stretchability (~500%), linear temperature response (*R*^2^ = 1) and fast time response (~332 ms) [150]. Majidi et al. [151] have developed thin-film curvature sensors composed of microfluidic channels filled with liquid metal (eGaIn) embedded in PDMS or Ecoflex substrates. The sensors can offer up to 1000% stretchability and measure both bending curvature and strain within the substrates with a gauge factor of 2 and a Young’s modulus of 0.1~1 Mpa. Roh et al. [87] have realized a transparent and patchable strain sensor that is made of a sandwich-like stacked piezoresistive nanohybrid film of single-wall carbon nanotubes (SWCNTs) and a conductive elastomeric composite of polyurethane (PU)-poly(3,4-ethylenedioxythiophene) polystyrenesulfonate (PEDOT:PSS). This sensor can offer stretchability of up to 100% and optical transparency of 62%, which can detect small strains on human skin (Figure 6c).

### 5.3. Skin Temperature Monitoring

**Figure 7:**
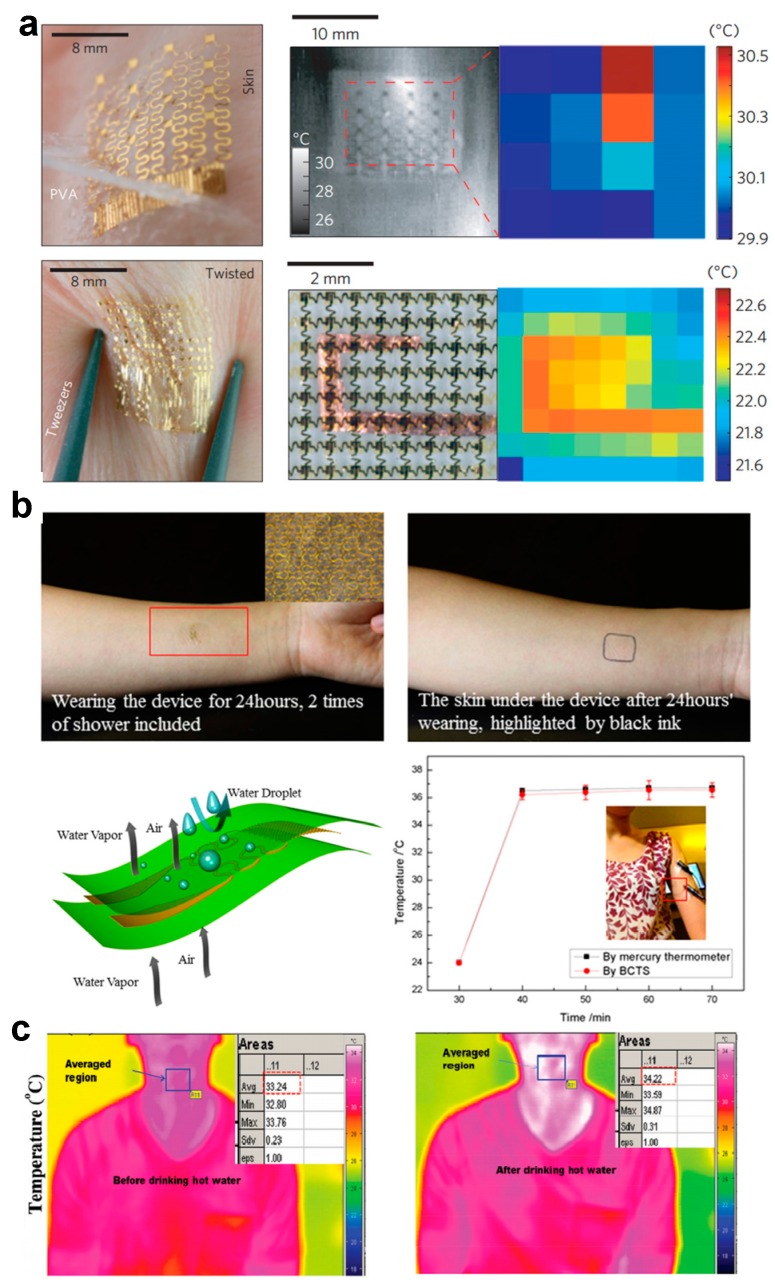
Examples of skin sensors for temepature measurement: (**a**) epidermal sensors that can monitor skin temperature using metallic and semiconductor sensors (Reprinted with permission from Ref. [159] Copyright 2013 Nature Publication Group); (**b**) breathable and stretchable temperature sensors (Reprinted with permission from Ref. [28] Copyright 2015 Nature Publication Group); and (**c**) transparent and stretchable temperature sensors (Reprinted with permission from Ref. [160] Copyright 2015 John Wiley and Sons).

Temperature sensors are essential components for many health monitoring systems to determine both physiological and psychological conditions associated with cardiovascular health, cognitive state and malignancy. Skin temperature sensors can be conformably attached to skin surface, and, thus, can accurately measure body temperature with minimized influence from the environmental temperature. Examples of skin temperature sensors include arrays of meander metal wires that determine body temperature through measurement of spatial mapping, and temperature mapping devices based on PIN diodes made of silicon nanomembranes [159] (Figure 7a). To improve capability of long-term integration without disturbing the functions of skin, skin temperature sensors can be integrated with breathable substrates made of porous, semipermeable PU films (Figure 7b). The entire sensor is permeable to air and waterproof. It can realize continuous body temperature measuring for up to 24 h [28]. Organic materials can also be used to construct temperature sensors. Trung et al. [160] have realized a resistive and gated temperature sensor array purely by elastic organic materials with stretchability of ~70% and sensitivity of ~1.34% resistance change per degree Celsius. The sensing layer of this device can be formed by imbedding conductive and graphene oxide nanosheets into an elastomeric PU matrix (Figure 7c).

### 5.4. Hydration Sensing

Accurately measurement of skin hydration levels is important for analyzing various diseases (e.g., dermatitis [161], psoriasis [162], eczema [163] and pruritus [164]) in the fields of dermatology and cosmetology, and evaluating factors (e.g., environmental [165], age [166], and hormone [167]) related to abnormal skin responses. In addition, hydration can also be used for assessing effectiveness of anti-aging treatment, moisturizing treatments and other medical therapies.

**Figure 8:**
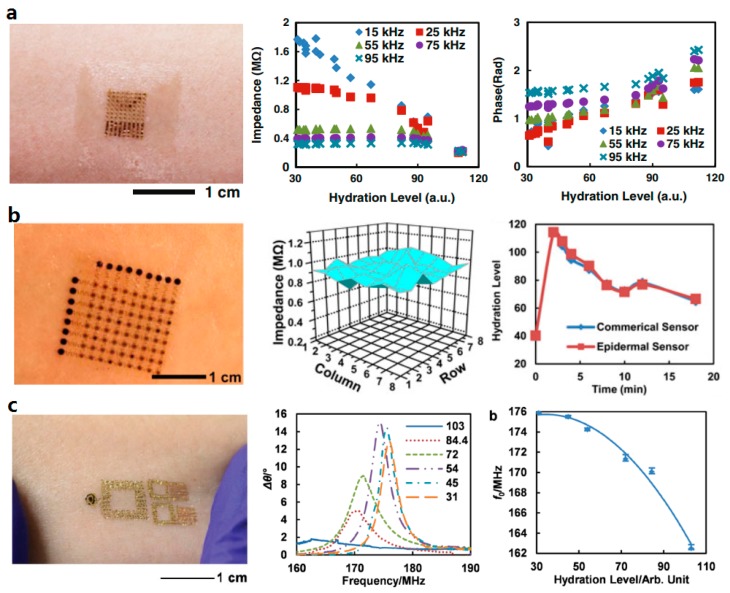
Examples of skin sensors used for hydration sensing: (**a**) epidermal sensor that can monitor biopotential on skin using metallic meshes (Reprinted with permission from Ref. [14] Copyright 2013 John Wiley and Sons); (**b**) hydration sensor that can conduct regional mapping based on the impedance detection (Reprinted with permission from Ref. [27] Copyright 2014 IEEE); and (**c**) hydration sensor capable of passive wireless detection (Reprinted with permission from Ref. [15] Copyright 2014 John Wiley and Sons).

Skin hydration can be determined by measurements of electrical impedance, thermal conductivity, spectroscopic property, and mechanical characteristic in conventional approaches. The application of epidermal electronic techniques gives hydration sensing many advantages over traditional methods. Huang et al. have realized several types of epidermal hydration sensors based on detection of skin electrical impedance. The sensors consist of two electrodes connecting with a data acquisition system, which provides alternating electrical current at frequencies between 1 and 100 kHz. The skin electronic impedance can be reflected by resulting attenuation and phase shift of the electrical current. Devices capable of conducting differential monitoring [14] (Figure 8a), regional mapping [27] (Figure 8b), and wireless sensing [15] (Figure 8c) have been developed based on the impedance detection. In addition, hydration can also be assessed through measurements of skin thermal conductivity, which can be determined by time response of skin to constant thermal energy input [159].

### 5.5. Biomolecule Analysis

**Figure 9:**
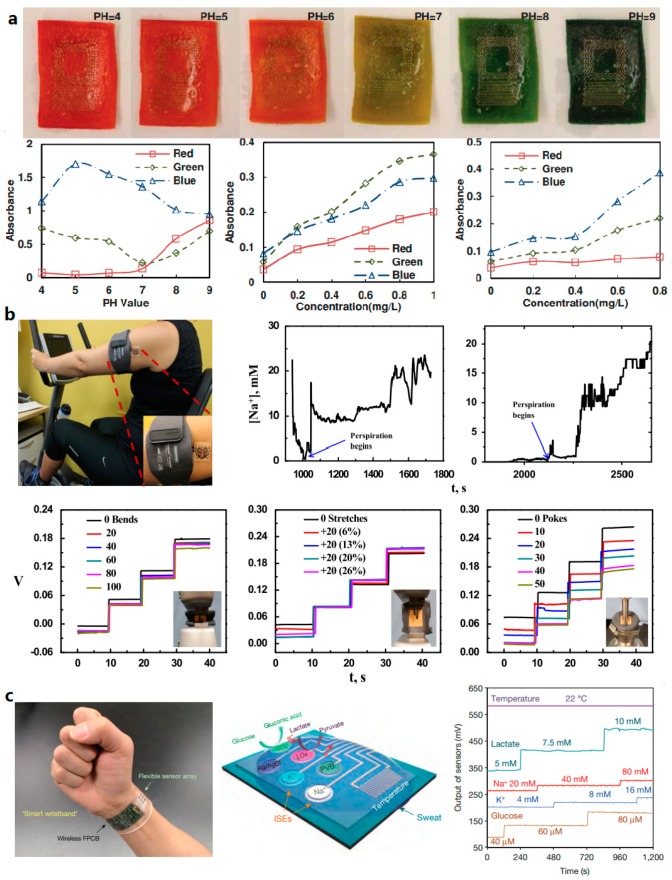
Examples of skin senosrs capable of conducting biomolecule analysis: (**a**) a skin sensor that can monitor biomolecules in sweat based on colorimetry approach (Reprinted with permission from Ref. [13] Copyright 2014 John Wiley and Sons); (**b**) a skin sensor that can monitor sodium in perspiration using electrochemical methods (Reprinted with permission from Ref. [175] Copyright 2014 Elsevier); and (**c**) a integrated system that can analyze multiple compositions in sweat simultaneously and selectively (Reprinted with permission from Ref. [12] Copyright 2016 Nature Publishing Group).

Flexible and stretchable skin sensors can be utilized for biomolecule analysis. Various biomolecules in sweat (e.g., sodium [12,168,169,170], potassium [12,169], ammonium [171,172], glucose [173], and lactate [12,174]) have been regarded as indicators for human physiological health. Huang et al. have explored materials and design strategies for integrating stretchable wireless sensors on porous sponge-like elastomeric substrates for epidermal analysis of biomolecules in sweat (Figure 9a). The porous substrates allow sweat collection through capillary forces, without need for complex microfluidic handling systems. Colorimetric measurement is achieved in the same system by introducing indicator compounds into the substrates for sensing specific components (OH^−^, H^+^, Cu^+^, and Fe^2+^) in sweat [13]. Bandodkar et al. [175] have developed an epidermal tattoo-like sensor using a bluetooth enabled wearable transceiver for real-time monitoring of sodium in human perspiration with concentration range of 0.1–100 mM. This sensor can withstand strain caused by bending, stretching and poking (Figure 9b). Gao et al. [12] have designed a fully integrated sensor array for in situ perspiration analysis, which can simultaneously and selectively measure sweat metabolites (e.g., glucose and lactate) and electrolytes (e.g., sodium and potassium ions) as well as the skin temperature for sensor calibration (Figure 9c).

### 5.6. Other Sensing

**Figure 10:**
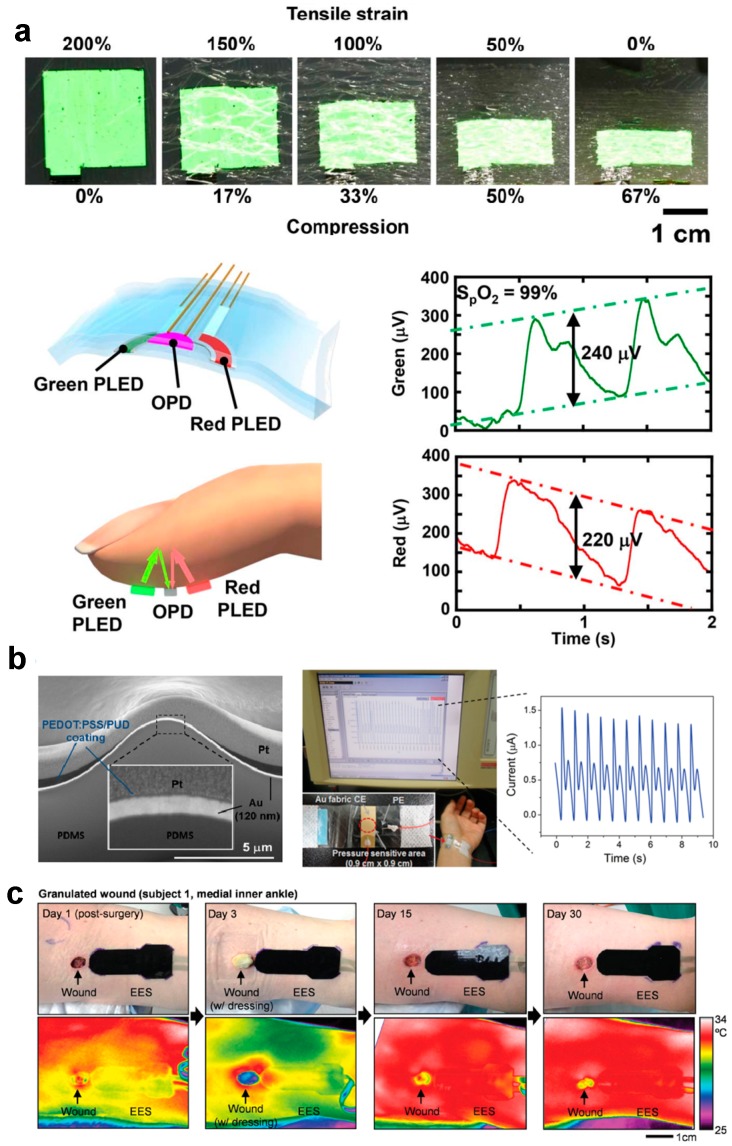
Skin sensors used for other sensing applications: (**a**) a skin sensor that can measure oxygen concentration of blood (Reprinted with permission from Ref [177] Copyright 2016 The Authors); (**b**) a stretchable resistive pressure sensor (Reprinted with permission from Ref [182] Copyright 2014 John Wiley and Sons); and (**c**) an epidermal electronics system that can monitor cutaneous wound healing (Reprinted with permission from Ref. [180] Copyright 2014 John Wiley and Sons).

Flexible and stretchable skin sensors also have other applications, including oximetry [21,176,177], pressure sensing [178,179] and wound healing monitoring [180,181]. For example, Yokota et al. [177] have developed optoelectronic skins integrated with OLED and organic photodetectors, which can measure the oxygen concentration of blood based on a photoplethysmogram (PPG) approach (Figure 10a). Choong et al. [182] have demonstrated a stretchable resistive pressure sensor within which the conductive electrode is built on the micro-pyramid PDMS arrays grafted with a PEDOT:PSS/PUD composite polymer. The sensor offers a pressure sensitivity of 10.3 kPa^−1^ when stretched by 40% (Figure 10b). Hattori et al. [180] have established an epidermal electronics system that can monitor cutaneous wound healing by recording time-dynamic temperature and thermal conductivity of skin. This system consists of metal traces with fractal and filamentary serpentine (FS) configurations, which can offer stretchability of ~30% (Figure 10c).

## 6. Conclusions and Perspectives

This paper reviews the mechanisms, materials, fabrication techniques, and the representative applications of flexible and stretchable skin sensors. These sensors are constructed by various intrinsic soft materials or stretchable thin film structures with applications in biophysiological signal measurement and activity tracking, offering improved precision and effectiveness of body integration. Flexible and stretchable skin sensors can collect massive amount of data associated with personal biomedical information and life-style. These data can be used to assist more specific diagnosis and effective treatment of disease, and can potentially be used to reveal the underlying connection among biomedical information, environmental effects, and various diseases.

Despite the rapid progress in skin sensors, development of flexible and stretchable skin sensors has encountered several critical issues such as power supplies and system complexity. Firstly, most of the devices mentioned above focus on sensing functions of the stretchable electronic devices. However, power supply, signal conditioning, data communication, and data storage still largely rely on bulky instruments or integrated circuits based on rigid substrates. Some researches tackle the issues of power supplies with stretchable batteries [48,183], piezoelectric generators [184,185], solar cells [186,187], and wireless power harvesting [188], showing promising future in replacing current bulky power sources with components that mechanically and geometrically match stretchable electronic devices. While for signal conditioning and data communication, and data storage, these functions can be realized by integrating multiple commercial-off-the-shelf components connected by flexible and stretchable interconnects. These systems can be best represented by the work of Xu et al. [44] who realized a complex measurement system to detect biopotential, acceleration, temperature and achieve signal processing and wireless data communication with an operational amplifier-based circuit and a voltage control oscillator. The sweat sensing system [12] mentioned in the previous section has also demonstrated the possibility of integrating flexible circuits made of commercial components with stretchable skin sensor for precise analysis of sweat contents. These comprehensive systems will lead to capability to assess multiple biophysiological signals to improve accuracy in diagnosis and treatment. Discrete stretchable electronic components based purely on thin film materials and stretchable structures have been demonstrated as signal amplifiers [189], logic circuits [190,191], oscillators [192], and nonvolatile resistive memory [193,194]. However, a fully stretchable and integrated system has not yet been achieved due to the challenges in the fabrication of different functional components, interconnection of transfer printed components, and low electronic performance as compared to conventional devices based on rigid materials. 

Furthermore, some fundamental knowledge of device mechanisms has not yet been well studied mechanically and electrically, and the interaction between the biological tissues and the flexible and stretchable skin sensors has not yet been well understood. For example, the electromagnetic properties of the stretchable structures such as serpentine, wavy, and out-of-plane buckling are largely unexplored, and the negative effects of the biological tissues to electromagnetic signal and optical signal have not yet been addressed to achieve optimized sensor performance. It can be expected that special properties offered by the sensor/skin interaction may be used to achieve more unique functions such as spontaneous actuation and transduction through skin motions, and the skin barrier functions may be overcome to allow analysis of biomolecules in blood and interstitial fluids using skin sensors. With more understanding of the fundamental knowledge of flexible and stretchable skin sensors, more sensing functions and powerful integrated systems may be developed based on the skin sensor platform, allowing revolutionary changes in the formats of continuous, long-term health monitoring devices to improve social health levels.
